# Trends of Exclusive Breastfeeding Practices and Its Determinants in Tanzania from 1999 to 2016

**DOI:** 10.3390/ijerph20206904

**Published:** 2023-10-10

**Authors:** Ola Farid Jahanpour, Jim Todd, Henry Mwambi, Elphas Luchemo Okango, Michael J. Mahande

**Affiliations:** 1 Department of Epidemiology and Biostatistics, Institute of Public Health, Kilimanjaro Christian Medical University College (KCMUCo), Moshi P.O. Box 2240, Tanzania; 2 Department of Population Health, London School of Hygiene and Tropical Medicine, London UK and National Institute for Medical Research, Mwanza P.O. Box 1708, Tanzania; 3 School of Mathematics, Statistics & Computer Science, University of KwaZulu-Natal, Pietermaritzburg 3209, South Africa; 4 Department of Mathematical Sciences, Strathmore University, Kitale P.O. Box 850-30200, Kenya

**Keywords:** exclusive breastfeeding, generalized linear mixed models, demographic health survey, secondary analysis, determinants, trend, disparities

## Abstract

**Introduction**: The benefits of exclusive breastfeeding (EBF) are widely reported. However, it is crucial to examine potential disparities in EBF practices across different regions of a country. Our study uses Tanzania demographic and health survey data to report on the trends of EBF across regions from 1999 to 2016, the patterns of the practice based on geographical location and socioeconomic status, and explores its determinants across the years. **Methods**: Descriptive statistics were used to establish the trends of EBF by geographical location and wealth quintile. A generalized linear mixed model was developed to incorporate both infant and maternal attributes as fixed covariates while considering enumeration areas and regions as clusters. The fitted model facilitated the estimation of EBF proportions at a regional level and identified key determinants influencing EBF practices across the survey periods. Moreover, we designed breastfeeding maps, visually depicting the performance of different regions throughout the surveys. **Results**: Across the various survey rounds, a notable regional variation in EBF practices was observed, with coastal regions generally exhibiting lower adherence to the practice. There was a linear trend between EBF and geographical residence (*p* < 0.05) and socioeconomic standing (*p* < 0.05) across the survey periods. Rural-dwelling women and those from the least affluent backgrounds consistently showcased a higher proportion of EBF. The prevalence of EBF declined as infants aged (*p* < 0.001), a trend consistent across all survey waves. The associations between maternal attributes and EBF practices displayed temporal variations. Furthermore, a correlation between exclusive breastfeeding and attributes linked to both regional disparities and enumeration areas was observed. The intra-cluster correlation ranged from 18% to 41.5% at the regional level and from 40% to 58.5% at the enumeration area level. **Conclusions**: While Tanzania’s progress in EBF practices is laudable, regional disparities persist, demanding targeted interventions. Sustaining achievements while addressing wealth-based disparities and the decline in EBF with infant age is vital. The study highlights the need for broad national strategies and localized investigations to understand and enhance EBF practices across different regions and socioeconomic contexts.

## 1. Introduction

The World Health Organization advocates for exclusive breastfeeding (EBF) up to six months of age, with infants receiving only breast milk along with prescribed medicines and vitamins [1]. EBF offers many advantages, including the easy accessibility of breast milk [2]. It is a vital source of nourishment, fostering optimal growth and development in infants [3,4]. Notably, it shields against hypothermia [5,6] and hypoglycemia—both significant contributors to early neonatal mortality. Moreover, breast milk bestows protection against infections [4,6,7], necrotizing enterocolitis, and sudden infant death syndrome [6].

In Tanzania, the practice of EBF has exhibited a consistent upward trajectory. In 1991/2, only 26% of infants under 6 months of age were EBF, a figure which surged to 59% by 2015 [8]. The nation has implemented a diverse array of structures, strategies, and interventions aimed at safeguarding, promoting, and supporting EBF. As early as 1973, the Tanzania Food and Nutrition Center was established [9], an independent institute entrusted with coordinating all nutritional endeavors within the country. The inaugural food and nutrition policy emerged in 1992. Notably, Tanzania has embraced internationally recommended measures and strategies, including the adoption of a baby-friendly hospital initiative in 1990 [9], adherence to the code of marketing of breastmilk substitutes in 1994 [9,10], and alignment with the global strategy for infants and young child feeding in 2003 [9]. The nation has further introduced numerous strategies to bolster the prevalence of EBF [11].

The determinants of EBF encompass a range of factors associated with both the infant and the mother. Infant-related determinants include factors such as sex [12,13], age [14,15,16,17], and whether born as a singleton or multiples [12]. Maternal determinants encompass age [12], educational level [12,14], marital status, and wealth quintile [12,13,18]. Additional determinants related to breastfeeding practices encompass the residential area, distinguishing between urban and rural contexts [6,11,13,15,18,19], regional disparities [15], and ecological distinctions [18].

The trajectory of the EBF proportion demonstrates considerable variation among countries, with trends ranging from upward surges [8,17] to declines [13], fluctuations [20], and relative stability [15]. Moreover, determinants of EBF may evolve over time [15,18], and the impact of certain characteristics on breastfeeding practices can shift over time [6,15,18]. Research has also highlighted disparities in EBF practices across diverse locations and ethnicities within a country [4,13,15,21,22,23]. Consequently, a national trend in EBF practices might not accurately reflect the dynamics at play in various regions. 

It remains uncertain whether Tanzania’s notable achievements in enhancing the proportion of exclusive breastfeeding (EBF) at the national level are equally manifested across all regions and social strata. Furthermore, the potential variations in determinants of EBF practices in Tanzania over the years have yet to be elucidated. 

The primary objective of this study is to shed light on the trends of EBF across distinct regions within Tanzania, spanning the period from 1999 to 2016. The study aims to uncover the pattern of EBF adoption based on different residential areas and wealth quintiles during this study time. Additionally, the study seeks to explore and elucidate the multifaceted determinants that have influenced the practice of EBF over this extended duration. This study is poised to provide stakeholders with invaluable historical insights into the trajectories of regional EBF practices, highlighting any performance discrepancies based on residential areas and wealth quintiles. By delving into the shifting determinants of EBF practices over the study period, the research offers a nuanced understanding of the factors driving these trends and potential variations. With this knowledge, stakeholders can formulate targeted interventions that effectively address regions requiring attention, disparities in residential areas, and nuanced considerations related to wealth-based dynamics. Such evidence-based insights have the potential to serve as a compass guiding future efforts in the promotion and support of EBF practices.

## 2. Materials and Methods

### 2.1. Study Design

A cross-sectional study utilized data from the Tanzania DHS spanning 1999, 2004/5, 2010, and 2015/16, all equipped with standard DHS datasets. The selection of 1999 as the baseline year is founded upon its proximity to the inauguration of the Millennium Development Goals (MDG) in 2000. Conversely, including the 2015/16 dataset corresponds to the culmination of MDG implementation and also serves as the most current data available during the analysis phase.

### 2.2. Study Area

The research encompasses the United Republic of Tanzania, including Tanzania Mainland and Zanzibar. The configuration of regions has evolved over time, with certain larger regions subdividing to create new ones. The population has steadily grown, rising from 23.1 million in 1988 to 45 million in 2012 [24]. While urbanization has witnessed rapid expansion since 1990, a significant majority of the populace still resides in rural areas. Notably, the pace of urbanization differs among various regions. Agriculture remains the primary livelihood for more than half of the population. Disparities in primary school enrollment rates are evident across the nation. The availability and distribution of healthcare facilities and personnel pose challenges, particularly pronounced in rural locales [24]. 

### 2.3. Data Source

Elaboration on the DHS surveys has been documented elsewhere [25]. The DHS surveys stratify the nation by geographic regions and urban/rural contexts. This is then followed employing a two-stage-cluster sampling technique. The initial stage involves the selection of a cluster (known as an enumeration area), followed by choosing a specific household within that cluster. Inhabitants of selected households who meet the inclusion criteria are then interviewed. The response rates were 98% in 1999, 99% in 2004/05, 99% in 2010, and 98% in 2015/16. The survey collects data on mothers, their infants, and feeding practices. This information was extracted for the purpose of this study.

### 2.4. Study Participants and Sampling Procedure

**Figure 1:**
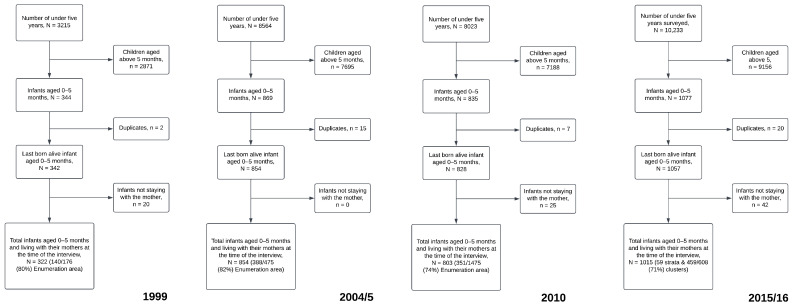
Flow chart of participants from the surveys (1999 to 2016).

Figure 1 illustrates the flow of participants throughout the survey rounds. The study cohort comprised mothers and their youngest infants residing with them during the interview. The range of infants aged 0–5 months included in the surveys spanned from 322 to 1015. These infants hailed from 80%, 82%, 74%, and 71% of all enumeration areas in 1999, 2004/5, 2010, and 2015/16, respectively (Figure 1).

### 2.5. Study Variables

#### 2.5.1. Dependent Variable

The outcome variable was consistently defined across all surveys. Infants exclusively breastfed in the 24 h preceding the interview were categorized as “Exclusively Breastfed” (1 “Yes”), while those who received substances other than prescribed medicines, oral rehydration solution, vitamins, and minerals were considered “Not Exclusively Breastfed” (0 “No”).

#### 2.5.2. Exposure Variables

Variables were chosen based on existing literature and data comparability across surveys. Infant-related variables included sex and age, while mother-related variables comprised age, area of residence, education level, employment status, radio and television usage frequency, and marital status. Infant sex was categorized as male or female, with age measured in months. Infant age was analyzed both categorically in descriptive analysis and continuously in modeling for determinants of EBF. The mother’s age was grouped as <18, 18–24, and 25+ years. The wealth quintile was divided into the lowest, low, middle, high, and highest quintiles. The area of residence was categorized as urban or rural. Education level was classified as no education, incomplete primary, complete primary/incomplete secondary, and complete secondary/higher. Employment status included categories such as working for family/someone else, self-employed, and not working. The frequency of radio listening, as well as television watching, was categorized as less than once a week, at least once a week, and almost every day. Marital status was grouped into never in union, widowed, divorced, no longer living together, and married/living with a partner. Antenatal clinic visits were categorized as 0–3 visits and 4 or more visits.

### 2.6. Statistical Methods

#### 2.6.1. Background Characteristics

For each survey, covariates were summarized using unweighted and weighted frequencies, proportions, and 95% confidence intervals. A significance level of *p* < 0.05 was applied to all analyses.

#### 2.6.2. Maps Development

QGIS (QGIS Development Team. “QGIS Geographic Information System.” Open Source Geospatial Foundation Project. Available: https://qgis.org (accessed on 21 January 2021)) was employed to create maps. Maps were generated for each survey, illustrating regions with EBF proportions below 25% of the interquartile range (IQR) median and regions exceeding 75% of the median IQR.

#### 2.6.3. The Trend of EBF Practices by Area of Residence and by Wealth Quintile

Two approaches were used to assess the trend of EBF practices across surveys. The first employed a Chi-square test to examine linear changes in EBF practices across surveys. Proportions of EBF were estimated for each year, considering survey characteristics (weight and clusters). The Chi-square test compared survey-based trends by area of residence and wealth quintile. The second approach utilized a generalized mixed linear model with varying slopes for the area of residence and wealth quintile for each survey.

#### 2.6.4. Model Selection for the Determinants of EBF across Surveys and Its Validation

To comprehend the determinants influencing EBF practices, a backward selection of variables was constructed. For each survey, the backward variables selection model encompassed infant-related and mother-related factors. Variables selected in at least one of the surveys were used in the final model. Marital status and the frequency of antenatal clinic visits were consistently excluded from the final model as they did not meet the selection criteria in any of the surveys. After the variables were selected, six models were compared for each survey, utilizing classical logistic regression and generalized linear mixed models, with variations in how infant age was treated. The models accounted for the survey nature of the data, considering the sampling weight and clusters. In the generalized mixed linear model, a binomial distribution was assumed, and a logit link was used. Random effects were applied to enumeration areas nested in regions from all surveys.

For each survey, six models were compared:

i. Classical logistic regression with the age of an infant as a categorical variable;
ii. Classical logistic regression with the age of an infant as a continuous variable;
iii. Generalized linear mixed model accounting for clustering at regional level with infant’s age treated as a categorical variable;
iv. Generalized linear mixed model accounting for clustering at regional level with infant’s age as a continuous variable;
v. Generalized linear mixed model accounting for enumeration area nested in regions with infant’s age treated as categorical;
vi. Generalized linear mixed model accounting for enumeration area nested in regions with infant’s age as a continuous variable.

Model selection was based on Akaike information criteria (AIC) scores. Additionally, a model incorporating the survey year as a fixed variable was examined to understand its contribution to changes in EBF proportions over the years. Two models were compared, one with a year of survey as a categorical variable and the other with the year of survey as a continuous variable. The model with the year of survey as a continuous variable performed better based on AIC scores and was treated as a separate model (model vii).

## 3. Results

### 3.1. Background Characteristics of the Participants from the Surveys

An overview of the characteristics of study participants across the different survey years is presented in Table 1. The proportion of infants exclusively breastfed increased from 31.8% (95% CI 22.5, 39.3%) in 1999 to 59% (95% CI 56%, 63%) in 2015/16. The distribution of infants across different age groups remained relatively consistent across the surveys, with approximately 10% to 20% of infants falling into each age category. Most mothers had completed primary or incomplete secondary education across all survey years. However, there was a significant shift in the 2015 survey, where the proportion of mothers who had completed secondary education increased to 10%, a notable increase compared to less than 1% in previous surveys. A smaller portion of mothers were not working compared to those working for a family member or someone else or self-employed. In 1999, 40% of deliveries took place in health facilities, and this percentage increased to 64% in 2015 (Table 1).

### 3.2. The Trend of EBF by Region across the Surveys

**Figure 2:**
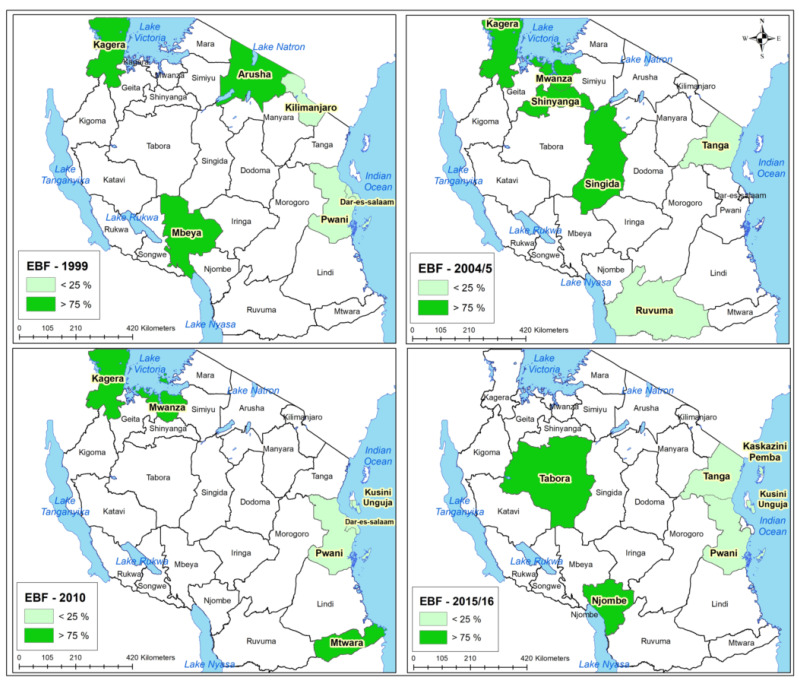
A map of Tanzania showing regions with the lowest and highest proportion of EBF from 1999 to 2016 (Top left 1999, top right 2004/5, bottom left 2010, bottom right 2015/16).

Figure 2 displays maps for each survey year, illustrating regions with EBF proportions above 75% of the median (darker green) and those below 25% (lighter green). In 1999, lower EBF proportions were observed in coastal and inland regions. By 2010, coastal areas and Zanzibar showed lower EBF proportions, unlike regions along Lake Victoria, which had favorable proportions except in 2015/16 (Figure 2).

### 3.3. Variation of EBF Based on the Area of Residence and the Wealth Quintile

**Figure 3:**
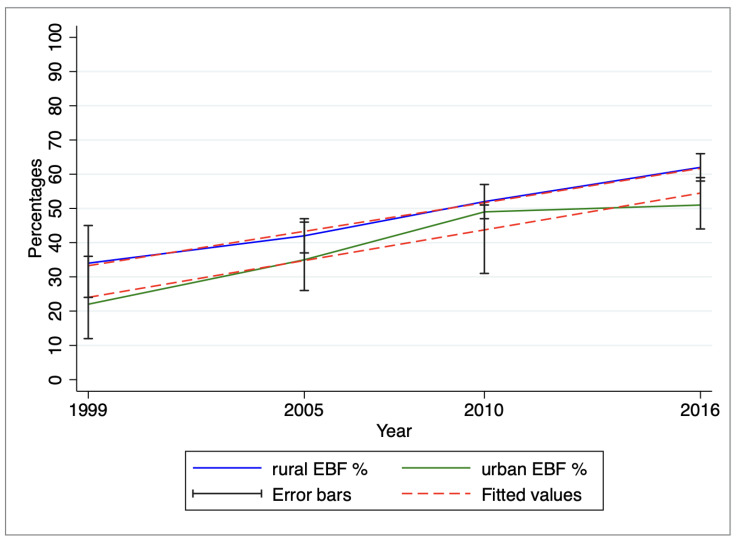
Variation of exclusive breastfeeding by area of residence from 1999 to 2016.

**Figure 4:**
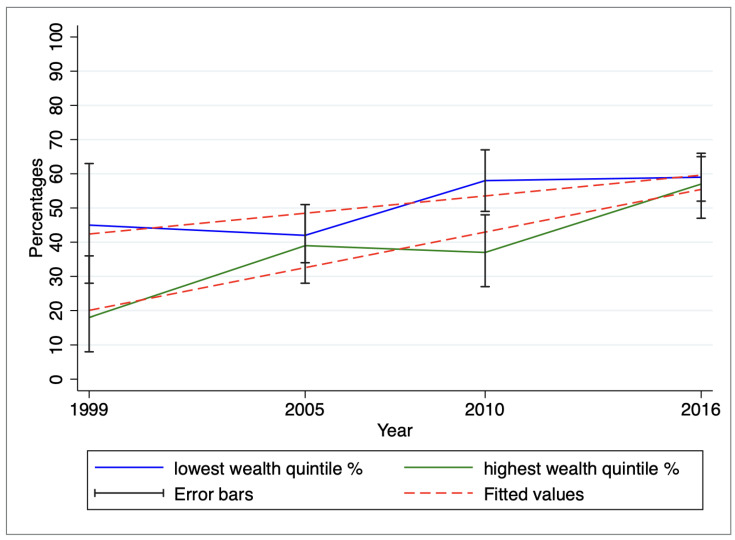
Variation of exclusive breastfeeding by wealth quintile from 1999 to 2016.

A generalized mixed linear model incorporating regional variability in slopes based on wealth and area of residence demonstrated a small variance of 0.03 or less within these specific regions (data not shown). Utilizing a Chi-square test, statistically significant linear trends in EBF were identified across the survey periods, stratified by area of residence (*p* < 0.001) and wealth quintile (*p* < 0.001). The graphical representation of these trends and slopes is illustrated in Figure 3 (by area of residence) and Figure 4 (by wealth quintile). 

The prevalence of EBF has consistently been higher among rural women compared to their urban counterparts, and this trend has shown a steady increase over time (refer to Figure 3). Moreover, EBF rates have exhibited higher and varying patterns among individuals belonging to the lowest wealth quintile. In contrast, EBF rates among those from the highest wealth quintile have either shown a modest increase or remained relatively stable. Notably, by the survey period 2015/16, the proportion of EBF among the lowest and highest wealth quintiles has nearly converged, as depicted in Figure 4.

### 3.4. Determinants of EBF across the Surveys

Table 2 provides an overview of the determinants of EBF utilizing a generalized mixed linear model, where enumeration areas (EAs) are nested within regions, representing the optimal performing model (model vi). Upon examining the adjusted model, a consistent trend emerged across all survey iterations, revealing a notable decline in EBF proportions as the infant’s age advanced. Notably, with each incremental month of infant age, the odds of practicing EBF exhibited a reduction ranging from 0.2 to 0.5, consistently yielding statistical significance (*p* < 0.001).

In 2004/5, the wealth quintile exhibited a statistically significant association with EBF practices, wherein both the lowest (reference group) and highest wealth quintile demonstrated higher odds of practicing EBF (*p* = 0.941). In 2015/16, the area of residence displayed a significant association with EBF, with rural settings being twice as likely to adopt EBF compared to urban settings (*p* = 0.035). Though not statistically significant in other survey years, rural areas generally tended to have higher EBF practices, except for 1999.

Based on the mother’s education level, those who were more likely to practice EBF in 1999 were those with no education (reference group); in 2004/5 were those who completed primary/incomplete secondary (*p* = 0.05) and completed secondary/higher (*p* = 0.002); in 2010 were those with complete secondary/higher education (*p* = 0.9); and in 2015/16 were those who completed primary/incomplete secondary (*p* = 0.038) and complete secondary/higher (*p* = 0.043). 

Mother’s working status was associated with EBF practices in 1999 and 2010. In 1999, compared to working for a family member/someone else (reference group), being self-employed increased the odds of practicing EBF (*p* = 0.025), but there was no difference for women who were not working (*p* = 0.792). In 2010, those not working (*p* = 0.022) and those who were self-employed (*p* = 0.223) were more likely to practice EBF.

The place of delivery did not exhibit any statistical association with EBF practices. Except for the 1999 survey, individuals who delivered at a health facility were more likely to practice EBF than those delivering at home.

Across all surveys, there was variability in EBF practices at both regional and enumeration area levels. The variability was greater at the enumeration area level compared to the regional level. In 1999, 58.5% of the variability in EBF practices could be attributed to factors related to enumeration areas, whereas in other surveys, this accounted for about 40% of the variability.

Controlling for other factors, when treating the year of the survey as a fixed variable (model vii), the odds of practicing EBF increased by 9% (95% CI 7–11%) from one survey to another (*p* < 0.001) (Data not shown).

## 4. Discussion

Tanzania has made commendable progress towards achieving universal EBF practices among infants under six months. By 2015/16, over half of mothers with infants under 6 months of age were practicing EBF. However, disparities persist across regions within the country, particularly in coastal and southern areas. The decline in EBF proportion as infants age remains consistent over the years. Mother-related characteristics’ contributions to EBF practices have shown variability, with lower wealth quintile, rural residence, education, and health facility delivery favoring EBF. While regional variabilities have decreased, significant variations persist at the enumeration area level since 2004/5.

Previous research has consistently highlighted the diverse prevalence of EBF practices across distinct regions within a country [4,13,15,21,22,23]. Notably, in Kenya, the coastal region displayed commendable EBF rates [22], whereas our study reveals that women residing in coastal and southern areas of Tanzania exhibit the lowest EBF proportions. Remarkably, our findings align with another comprehensive Tanzania analysis [10]. Despite Tanzania’s implementation of baby-friendly health initiatives [11], variations in healthcare facility quality among regions might contribute to the observed differences in EBF rates. Furthermore, discrepancies in HIV prevalence rates across time and regions could contribute to the varying EBF patterns [10,26]. The influence of cultural practices, which vary geographically, cannot be overlooked, impacting the adoption of EBF [5,10,23,27,28]. To gain deeper insights into the factors driving EBF adoption, conducting studies in smaller geographical areas is crucial. Effective interventions to enhance EBF should be strategically tailored to regions that are lagging behind, ensuring a targeted and impactful approach.

The relationship between the area of residence and EBF practices lacks consistency in the existing literature [11,19,22,26]. However, it is noteworthy that access to health facilities among urban residents has been associated with a higher proportion of EBF [19]. The substantial prevalence of EBF among women in rural settings can be attributed to cultural norms that promote and facilitate EBF. While breastfeeding is ultimately an individual decision, it is greatly influenced by the surrounding family and community dynamics [4,29]. Effective interventions should focus on promoting, safeguarding, and endorsing breastfeeding practices within urban environments. Simultaneously, enhancing the provision of high-quality healthcare services in rural areas is crucial to further strengthening EBF adoption. 

Consistent with findings from other investigations [6,15], our study underscores that women with lower wealth quintiles tend to embrace EBF more than those from higher wealth quintiles. In Tanzania, where breastfeeding is deeply ingrained in cultural norms, women with fewer alternatives are more inclined to opt for EBF. Research indicates that associating alternative feeding practices with elevated social status can lead to decreased EBF rates [18,23,30]. As a nation’s prosperity grows, there is a propensity for women to choose not to breastfeed. As Tanzania’s economy transitions toward a lower-middle-income status, it is crucial to maintain the strides made in promoting EBF thus far.

Interestingly, our study unveils that mothers from wealthier backgrounds are adopting EBF at an accelerated pace compared to those from lower wealth quintiles, in line with prior research [6]. This phenomenon could be attributed to increased access to knowledge regarding the benefits of EBF and improved facilities, such as conducive office environments and refrigeration for storing breast milk, which facilitate adherence to EBF practices. Conversely, women from lower wealth quintiles may contend with social and economic constraints that hinder sustained EBF [3,31]. Consequently, Tanzania must exercise caution to prevent the potential reversal of this trend, whereby women from the highest wealth quintiles breastfeed for longer periods than their counterparts from the lowest wealth quintiles, a trend observed elsewhere [6,22,23].

This study reveals a consistent decline in the proportion of EBF as infants age, a pattern that persists across all surveyed periods and regions. Despite initial variations in EBF proportions at birth, the observed decline remains uniform. To enhance EBF proportions and capitalize on its benefits, Tanzania should focus on curbing the practice of discontinuing EBF as infants grow older. Such efforts are attainable, as evidenced by successful strategies employed in countries like Nepal [18]. Implementing multifaceted interventions that address factors contributing to the cessation of EBF, such as misconceptions about milk sufficiency and managing common infant fussiness, could effectively counter this trend [4].

Various surveys have explored the connection between a mother’s level of education and their adoption of EBF practices. However, the existing literature presents diverse perspectives on the role of a mother’s education in EBF [13,21,22,31,32,33,34]. Crucially, a mother’s education level can serve as a proxy for several underlying factors. For instance, it can influence positive health-seeking behaviors [21], offer access to educational resources that promote EBF [4], and impact a woman’s employment status. In this study, an interesting finding emerges: unemployed or self-employed mothers are more inclined to practice EBF. This observation raises the possibility that the current policy supporting EBF among working women in Tanzania might be inadequate, either in terms of its duration or in its reach to a limited segment of the female population. It is worth noting that ensuring optimal breastfeeding should not solely be the mother’s responsibility [4]. Fathers, society at large, and governmental entities must collaborate to create an enabling environment that supports and empowers mothers to practice EBF. Recognizing the broader societal context and involving multiple stakeholders is vital for fostering successful EBF practices.

Although not statistically significant, this study reveals that, over the years, women who give birth at healthcare facilities are more inclined to practice EBF than their counterparts. This presents a valuable opportunity for targeted interventions. A pivotal aspect of enhancing EBF adoption among those utilizing healthcare facilities is the quality of counseling they receive [30,31,35,36]. The research underscores that not all mothers have received the necessary counseling to facilitate EBF adoption [3,23,31,37]. Inadequate training on EBF policy [26] might contribute to this gap. Moreover, limited financial support for EBF interventions in Tanzania [10] could lead to insufficient ongoing training for providers regarding the benefits of EBF and its practical implementation. It’s plausible that the shortage of adequate healthcare facilities in the country hampers the anticipated impact of facility-based deliveries on EBF practices [24]. It is imperative to address the availability and quality of healthcare facilities as a cornerstone of promoting EBF. By focusing on enhancing the counseling quality, training, and overall infrastructure within healthcare settings, the potential for boosting EBF practices can be maximized.

The implementation and expansion of interventions and strategies on a national scale aimed at supporting, promoting, and safeguarding EBF practices across Tanzania may have contributed to a reduction in the variability of EBF practices at both regional and enumeration area levels. This, in turn, could have played a role in the overall observed increase in the proportion of EBF over the years. Nonetheless, it’s important to note that this study identifies persistent disparities and discrepancies in EBF practices across different regions and enumeration areas. These findings underline the continued need for localized studies that delve into the intricate dynamics influencing EBF practices within smaller geographical areas. Such studies are vital for gaining a comprehensive understanding of the unique factors at play and determining targeted approaches to address them [4,6]. By conducting more focused research at the local level, policymakers and stakeholders can tailor interventions to tackle the specific challenges and barriers faced by different communities, thus further enhancing the promotion and adoption of EBF practices.

The policy implications of the findings from this study are as follows:

1. Region-specific interventions: Given the disparities in EBF practices across regions, it’s crucial to design region-specific interventions to address the lagging regions, particularly those along the coast. These interventions should be tailored to the cultural and socioeconomic contexts of each region, focusing on raising awareness about the benefits of EBF and providing adequate support to mothers. This calls for localized studies to understand the unique factors influencing EBF adoption. This will enable the development of targeted interventions that consider specific local dynamics;
2. Age-Specific EBF Support: Addressing the consistent drop in EBF proportions as infants age requires age-specific support mechanisms. Establish comprehensive educational programs that provide mothers, and society in general, with knowledge and skills to sustain EBF as infants grow, tackling challenges such as milk insufficiency perceptions and infant fussiness. The education program should be integrated into the extended program of immunization, as it often provides an opportunity for mothers and caregivers to visit healthcare facilities or interact with healthcare providers;
3. Urban EBF Promotion: Urban areas are also experiencing challenges in EBF adoption. Policy efforts should target urban populations with campaigns emphasizing the importance of EBF, dispelling misconceptions, and providing accessible resources for urban mothers to facilitate EBF practices amidst busy lifestyles;
4. Wealth-Targeted Programs: While wealthier mothers are catching up with EBF adoption, efforts should be made to prevent the reversal of this pattern. Targeted programs should be implemented that ensure accessibility to EBF information, counseling, and support for mothers across all income levels. This can prevent a scenario where wealthier mothers breastfeed for longer durations, leaving economically disadvantaged mothers at a disadvantage;
5. Sustaining EBF with Economic Growth: As Tanzania’s economy advances, efforts should be made to prevent the decline in EBF adoption observed in some wealthier segments. There is a need to establish breastfeeding-friendly workplaces, ensuring that working mothers have the support and facilities needed to continue EBF while pursuing their careers;
6. Quality Healthcare Facilities: The positive association between EBF and delivering at health facilities suggests the need for strengthening healthcare facilities’ role in EBF promotion. Healthcare providers should receive consistent training on EBF counseling to ensure accurate and reliable guidance to mothers. Enhancing the quality and accessibility of healthcare facilities can contribute to higher EBF rates.

### Limitations and Strengths of the Study

The study’s cross-sectional design limits the ability to establish causal relationships between determinant factors and EBF practices. It provides associations but cannot prove cause and effect. Also, using interviews for data collection may have introduced recall biases and socially desirable responses. Using the 24-h recall method to establish EBF practices has the potential to lead to an overestimation. A small sample size of infants across the surveys might have influenced the association of important independent variables with EBF practices. However, the sample size of infants involved in the surveys increased over the years, and infants were sampled from at least 70% of the enumeration areas. The analysis relies on the 2015/16 DHS dataset, and community patterns may have changed since then. More recent data would provide a more accurate picture of current EBF practices.

DHS employs rigorous sampling techniques and well-crafted tools, ensuring comprehensive data collection conducted by well-trained professionals. Consistently high response rates (>98%) across surveys underscore the robustness and generalizability of our findings to the entire nation. This study offers a unique contribution by juxtaposing four surveys spanning 1999 to 2015/16, elucidating trends across regions and delineating patterns based on residential areas and wealth indices. Moreover, it delves into the evolving determinants of EBF in Tanzania, a novel endeavor. This comparative analysis not only provides insights for future interventions but also establishes a benchmark for forthcoming evaluations. Utilizing a mixed model enhances the accuracy of determinant estimates, establishes regional and enumeration area correlations, and offers methodological insights pertinent to analyzing hierarchical DHS data.

## 5. Conclusions

Disparities persist in EBF practices across various regions of Tanzania. Coastal and urban areas lag, while EBF prevalence is more prominent among those with lower wealth indices. Notably, the proportion of EBF has increased overall; however, the consistent decline in EBF as infants age remains unchanged from 1999 to 2016. Noteworthy variations in EBF exist regionally and within enumeration areas, underscoring the need for targeted interventions. Coastal regions require particular attention while sustaining progress in other areas is imperative. Urban settings necessitate tailored interventions to promote EBF. Vigilance is crucial to retain achievements as the nation’s economy advances. Deeper insights into EBF adaptation demand localized studies in smaller geographic zones and finer infant age brackets.

## Figures and Tables

**Table 1:** Background characteristics of the participants across the surveys.

	1999 (N = 322)				2004/5 (N = 854)				2010 (N = 803)				2015/16 (N = 1015)			
	Overall Total (a)		Yes EBF (b)		Overall Total (a)		Yes EBF (b)		Overall Total (a)		Yes EBF (b)		Overall Total (a)		Yes EBF (b)	
	n	%	Row %	95% CI	n	%	Row %	95% CI	n	%	Row %	95% CI	n	%	Row %	95% CI
**Characteristics**																
EBF Proportion			31.80%	(22.5–39.3)			41	[36.8, 45.4]			49.9	[45.6, 54.2]			59.2	[55.7, 62.7]
Infant’s characteristics																
Sex of an infant																
Male	161	50	25.5	[17.2, 36.0]	417	48.8	42.5	[36.4, 48.9]	398	49.6	50.2	[44.4, 56.0]	507	50	59	[54.2, 63.7]
Female	161	50	38	[26.5, 51.1]	437	51.2	39.6	[34.1, 45.3]	405	50.4	49.6	[43.5, 55.7]	508	50	59.4	[54.4, 64.3]
Infant’s age (month)				**				**				**				**
0	37	11.5	53.9	[31.7, 74.7]	83	9.7	76.5	[65.2, 85.0]	80	10	86.2	[75.1, 92.9]	194	19.1	89.4	[83.7, 93.3]
1	60	18.6	59.6	[42.6, 74.6]	161	18.9	63.8	[54.9, 71.9]	162	20.2	78.3	[68.9, 85.4]	184	18.1	78.4	[71.1, 84.2]
2	56	17.4	29.6	[17.0, 46.4]	173	20.3	49.4	[40.5, 58.3]	146	18.2	57.9	[47.9, 67.2]	175	17.2	63.4	[54.9, 71.2]
3	63	19.6	21.3	[10.9, 37.5]	149	17.4	33.4	[24.5, 43.7]	128	15.9	44.2	[34.4, 54.5]	159	15.7	53.4	[44.5, 62.0]
4	57	17.7	15.3	[5.0, 38.1]	142	16.6	17.6	[11.1, 27.0]	145	18.1	26.3	[18.6, 35.8]	181	17.8	33	[25.1, 41.9]
5	49	15.2	15.7	[4.4, 42.9]	146	17.1	10	[5.4, 17.8]	142	17.7	20	[14.0, 27.7]	122	12	18.1	[11.5, 27.3]
Mother’s characteristics																
Mother’s age (years)																
Less than 18	18	5.6	40.1	[15.7, 70.6]	37	4.3	42.2	[24.5, 62.2]	39	4.9	57	[41.8, 70.9]	49	4.8	49.6	[32.7, 66.6]
18–24	110	34.2	27.8	[18.1, 40.0]	297	34.8	35.1	[28.6, 42.2]	283	35.2	49.5	[42.7, 56.3]	411	40.5	58.4	[52.8, 63.9]
25+	194	60.2	33.6	[22.9, 46.3]	520	60.9	44.6	[39.2, 50.2]	481	59.9	49.5	[44.1, 54.9]	555	54.7	60.8	[55.7, 65.7]
Current marital status																
Never in union/widowed/divorced/no longer living together	46	14.3	23.3	[11.0, 42.7]	90	10.5	31.1	[21.3, 43.0]	108	13.4	43.4	[33.6, 53.8]	147	14.5	57.7	[48.4, 66.5]
Married/living with partner	276	85.7	33.4	[24.0, 44.3]	764	89.5	42.3	[37.7, 47.0]	695	86.6	51	[46.0, 56.0]	868	85.5	59.5	[55.7, 63.2]
Wealth quintile												*				
Lowest	89	27.6	45.1	[28.4, 63.0]	176	20.6	42	[33.8, 50.7]	156	19.4	58.3	[49.2, 66.8]	258	25.4	58.9	[51.6, 65.8]
Low	39	12.1	20.9	[7.4, 46.4]	154	18	45.2	[36.5, 54.3]	190	23.7	54	[44.8, 63.0]	209	20.6	63	[54.5, 70.8]
Middle	57	17.7	31.9	[19.0, 48.3]	187	21.9	37.7	[30.6, 45.4]	178	22.2	51.5	[42.3, 60.6]	179	17.6	58.7	[50.5, 66.4]
High	54	16.8	39	[24.1, 56.3]	192	22.5	41.8	[32.6, 51.6]	177	22	42.5	[33.8, 51.6]	214	21.1	58.1	[49.3, 66.3]
Highest	83	25.8	17.9	[8.0, 35.2]	145	17	38.9	[28.5, 50.5]	102	12.7	37	[26.7, 48.7]	155	15.3	56.8	[47.2, 65.9]
Mother’s residence												*				*
Urban	77	23.9	21.9	[12.4, 35.7]	145	17	35.3	[25.9, 46.0]	135	16.8	40.3	[30.9, 50.4]	243	23.9	51.4	[44.0, 58.7]
Rural	245	76.1	34	[24.3, 45.2]	709	83	42.3	[37.6, 47.1]	668	83.2	52.2	[47.3, 57.0]	772	76.1	62.2	[58.2, 66.1]
Education attainment																
No education	92	28.6	39.7	[21.8, 60.8]	210	24.6	41	[32.4, 50.2]	207	25.8	50.6	[41.9, 59.3]	201	19.8	55.6	[47.9, 63.1]
Incomplete primary	50	15.5	28.7	[15.2, 47.6]	161	18.9	34.5	[25.1, 45.4]	138	17.2	50.1	[39.7, 60.6]	135	13.3	49.2	[40.4, 58.1]
Complete primary/incomplete secondary	178	55.3	28.3	[20.3, 38.0]	469	54.9	41.7	[36.6, 47.0]	453	56.4	49.7	[44.0, 55.5]	574	56.6	61.5	[56.5, 66.3]
Complete secondary/higher	2	0.6	0		14	1.6	79.9	[50.9, 93.9]	5	0.6	21.7	[2.8, 73.0]	105	10.3	65.8	[54.7, 75.5]
Who respondent works for								*								
For family member/Someone else	131	40.7	27.8	[18.9, 38.8]	592	69.3	43.9	[39.3, 48.5]	228	28.4	48.4	[40.8, 56.0]	362	35.7	62	[56.0, 67.6]
Self-employed	97	30.1	39.3	[26.9, 53.4]	125	14.6	27.6	[17.7, 40.3]	435	54.2	48.9	[43.0, 54.9]	410	40.4	61.5	[55.6, 67.0]
Not working	94	29.2	32.7	[16.3, 54.9]	137	16	33.3	[22.4, 46.3]	140	17.4	57.7	[47.2, 67.6]	243	23.9	51.3	[43.7, 58.9]
Frequency of listening to radio																
Not at all	110	34.2	40.3	[30.3, 51.2]	206	24.1	49.8	[42.1, 57.5]	263	32.8	55.8	[48.6, 62.7]	276	27.2	60.7	[53.8, 67.2]
Less than once a week	125	38.8	23	[13.5, 36.4]	105	12.3	35.8	[26.4, 46.4]	124	15.4	48.5	[37.4, 59.8]	359	35.4	58.5	[52.2, 64.6]
At least once a week	14	4.3	8.8	[1.6, 36.1]	172	20.1	36.1	[27.9, 45.3]	156	19.4	43.6	[34.4, 53.3]	380	37.4	58.8	[52.6, 64.7]
Almost everyday	73	22.7	39.3	[20.4, 62.1]	371	43.4	39.3	[32.9, 46.1]	260	32.4	47.7	[39.8, 55.7]				
Frequency of watching television								*				*				
Not at all	249	77.3	33.6	[24.1, 44.6]	677	79.3	44	[39.3, 48.7]	617	76.8	52.6	[47.7, 57.5]	597	58.8	60.1	[55.5, 64.5]
Less than once a week	54	16.8	25.3	[9.9, 51.2]	77	9	17.8	[10.0, 29.8]	89	11.1	46.4	[33.8, 59.6]	240	23.6	59.2	[51.7, 66.3]
At least once a week	2	0.6	0		63	7.4	32.2	[18.7, 49.5]	42	5.2	27.6	[14.9, 45.2]	178	17.5	56	[47.4, 64.2]
Almost everyday	17	5.3	1.2	[0.1, 9.0]	37	4.3	53.3	[28.4, 76.7]	55	6.8	39.5	[23.2, 58.5]				
Place of delivery				*												
Home	193	59.9	40.3	[30.5, 50.9]	456	53.4	41.2	[35.5, 47.1]	398	49.6	50.6	[43.8, 57.5]	358	35.3	59	[53.2, 64.7]
Health facility	129	40.1	20.2	[11.5, 33.0]	398	46.6	40.8	[34.4, 47.4]	405	50.4	49.2	[44.2, 54.2]	657	64.7	59.3	[54.7, 63.8]

a: unweighted, b: weighted, ** <0.0001, * <0.05.

**Table 2:** Factors associated with exclusive breastfeeding with enumeration areas nested in regions as random factors across the surveys.

	1999				2004/5				2010				2015/16			
	COR (95% CI)	*p*-Value	AOR (95% CI)	*p*-Value	COR (95% CI)	*p*-Value	AOR (95% CI)	*p*-Value	COR (95% CI)	*p*-Value	AOR (95% CI)	*p*-Value	COR (95% CI)	*p*-Value	AOR (95% CI)	*p*-Value
**Characteristics**																
Infant’s characteristics																
Sex of an infant																
female	1		1		1		1		1		1		1		1	
male	0.7 (0.4, 1.4)	0.358	0.6 (0.2, 1.4)	0.203	1.1 (0.8, 1.6)	0.45	1.3 (0.8, 1.9)	0.267	1.1 (0.8, 1.6)	0.483	1.2 (0.8, 1.8)	0.483	1.03 (0.8, 1.4)	0.869	0.9 (0.6, 1.3)	0.607
Infant’s age (month)	0.4 (0.3, 0.5)	<0.001	0.3 (0.2, 0.5)	<0.001	0.4 (0.4, 0.5)	<0.001	0.4 (0.3, 0, 5)	<0.001	0.4 (0.3, 0.5)	<0.0001	0.4 (0.3, 0.4)	<0.001	0.4 (0.3, 0.4)	<0.0001	0.4 (0.3, 0.4)	<0.0001
Mother’s characteristics																
Mother’s age (years)																
less than 18	1		1		1		1		1		1		1		1	
18–24	0.5 (0.1, 1.97)	0.323	0.3 (0.03, 2.2)	0.218	0.7 (0.3, 1.5)	0.321	0.9 (0.3, 2.7)	0.833	0.4 (0.2, 0.9)	0.022	0.4 (0.2, 1.2)	0.096	1.8 (0.9, 3.5)	0.106	1.3 (0.5, 3.2)	0.549
25+	0.6 (0.2, 2.3)	0.441	0.3 (0.03, 2.0)	0.198	1 (0.4, 2.2)	0.956	1.5 (0.5, 4.4)	0.481	0.4 (0.2, 0.9)	0.027	0.5 (0.2, 1.4)	0.187	2 (1, 3.9)	0.047	2.0 (0.8, 4.9)	0.114
Wealth quintile																
lowest	1		1		1		1		1		1		1		1	
low	0.3 (0.1, 0.8)	0.018	0.2 (0.04, 0.9)	0.04	1.03 (0.6, 1.8)	0.908	0.7 (0.4, 1.4)	0.361	1 (0.6, 1.6)	0.868	0.7 (0.4, 1.4)	0.359	1.3 (0.8, 2)	0.311	1 (0.6, 1.9)	0.905
middle	0.7 (0.31.6)	0.396	0.4 (0.1, 1.6)	0.222	0.6 (0.4, 1)	0.047	0.5 (0.3, 0.9)	0.034	0.8 (0.5, 1.3)	0.321	0.7 (0.4, 1.5)	0.366	1.2 (0.7, 1.9)	0.541	1.3 (0.7, 2.5)	0.415
high	0.9 (0.4, 2.4)	0.913	1.3 (0.3, 5.1)	0.695	1.1 (0.6, 1.8)	0.842	0.8 (0.4, 1.8)	0.725	0.5 (0.3, 0.9)	0.014	0.5 (0.2, 1.2)	0.125	1.3 (0.8, 2.1)	0.331	1.4 (0.7, 3)	0.317
highest	0.4 (0.1, 1.1)	0.085	0.8 (0.1, 5.5)	0.797	0.9 (0.5, 1.7)	0.725	1.03 (0.4, 2.9)	0.941	0.4 (0.2, 0.9)	0.02	0.5 (0.1, 1.5)	0.196	1.4 (0.8, 2.7)	0.232	1.4 (0.5, 3.9)	0.581
Mother’s residence																
urban	1		1		1		1		1		1		1		1	
rural	0.6 (0.2, 1.6)	0.288	0.9 (0.1, 5.3)	0.875	0.8 (0.4, 1.3)	0.299	1.6 (0.8, 3.5)	0.211	1.6 (0.9, 2.7)	0.093	1.5 (0.6, 3.6)	0.364	0.7 (0.5, 1.1)	0.165	2.1 (0.1, 4.2)	0.035
Education attainment																
no education	1		1		1		1		1		1		1		1	
incomplete primary	0.4 (0.1, 1.1)	0.063	0.1 (0.03, 0.7)	0.017	0.7 (0.4, 1.3)	0.294	0.7 (0.4, 1.5)	0.395	0.7 (0.4, 1.2)	0.202	0.5 (0.3, 1.1)	0.088	0.8 (0.5, 1.4)	0.477	1.1 (0.5, 2.2)	0.834
complete primary/incomplete secondary	0.5 (0.2, 0.99)	0.048	0.2 (0.1, 0.7)	0.011	1.2 (0.8, 1.8)	0.364	1.7 (0.9, 2.9)	0.05	0.8 (0.6, 1.3)	0.404	0.9 (0.5, 1.6)	0.748	1.5 (1, 2.3)	0.038	1.8 (1.03, 3.2)	0.038
complete secondary/higher					9 (1.8, 46.3)	0.008	12.2 (1.4, 103.1)	0.022	0.3 (0.02, 5.1)	0.416	1.3 (0.02, 80.3)	0.9	1.9 (1, 3.5)	0.059	2.6 (1.03, 6.3)	0.043
Working status																
For family member/Someone else	1		1		1		1		1		1		1		1	
Self-employed	2.2 (0.99, 4.7)	0.054	3.5 (1.2, 10.5)	0.025	0.5 (0.3, 1)	0.046	0.7 (0.3, 1.6)	0.393	1.2 (0.8, 1.8)	0.419	1.4 (0.8, 2.4)	0.223	0.7 (0.5, 1.1)	0.178	1.1 (0.7, 1.7)	0.742
Not working	0.99 (0.4, 2.3)	0.979	0.8 (0.2, 3)	0.792	1.1 (0.6, 2)	0.742	1.5 (0.7, 3.2)	0.276	1.9 (1, 3.7)	0.044	2.7 (1.2, 6.4)	0.022	1.03 (0.7, 1.5)	0.881	0.7 (0.4, 1.3)	0.258
Frequency of listening to radio																
not at all	1		1		1		1		1		1		1		1	
less than once a week	0.4 (0.2, 0.8)	0.015	0.2 (0.1, 0.6)	0.004	0.7 (0.5, 1.1)	0.12	0.6 (0.3, 1.0)	0.07	0.7 (0.4, 1.2)	0.206	0.5 (0.3, 1)	0.068	1.1 (0.7, 1.6)	0.699	1.1 (0.6, 1.9)	0.779
At least once a week	0.2 (0.03, 1.8)	0.164	0.2 (0.01, 3.3)	0.245	0.5 (0.3, 0.9)	0.016	0.4 (0.2, 0.7)	0.003	0.6 (0.4, 0.9)	0.048	0.6 (0.3, 1.1)	0.079	1 (0.7, 1.5)	0.977	0.7 (0.4, 1.3)	0.297
Almost everyday	1.7 (0.7, 4.3)	0.236	0.7 (0.2, 3)	0.603	0.7 (0.4, 1.2)	0.206	1 (0.5, 2)	0.939	0.6 (0.4, 1)	0.052	0.6 (0.4, 1.2)	0.173				
Frequency of watching television																
not at all	1		1		1		1		1		1		1		1	
less than once a week	1.1 (0.4, 2.8)	0.871	2.7 (0.6, 11.9)	0.204	0.3 (0.1, 0.5)	<0.001	0.2 (0.1, 0.6)	0.001	0.6 (0.4, 1.1)	0.11	1.2 (0.6, 2.5)	0.639	1.01 (0.7, 1.5)	0.927	1 (0.6, 1.7)	0.984
At least once a week					0.7 (0.4, 1.5)	0.366	0.8 (0.3, 2)	0.571	0.2 (0.1, 0.6)	0.001	0.4 (0.1, 1.2)	0.105	1.2 (0.7, 1.9)	0.523	1.3 (0.6, 2.7)	0.565
Almost everyday	0.02 (9.2 × 10^−6^, 52.6)	0.336	0.02 (6.3 × 10^−6^, 77.4)	0.36	1.7 (0.6, 4.9)	0.292	1.5 (0.3, 6.4)	0.587	0.8 (0.3, 1.9)	0.607	0.96 (0.3, 3.2)	0.953				
Place of delivery																
Health facility	1		1		1		1		1		1		1		1	
Home	2.8 (1.3, 6)	0.011	2.7 (0.8, 9)	0.098	0.9 (0.6, 1.2)	0.426	0.7 (0.4, 1.1)	0.08	0.9 (0.6, 1.3)	0.671	0.6 (0.4, 1)	0.069	0.8 (0.6, 1.2)	0.271	0.7 (0.4, 1.1)	0.135
Random factors (*p*-value)				<0.0001			<0.0001				<0.0001					<0.0001
Intracluster correlation (ICC) Regional			41.5% (95% CI: 19.3%, 67.9%)				26% (14%, 43%)				18% (8%, 36%)				20% (10%, 36%)	
ICC EA			58.5% (31.7%, 81.1%)				40% (25%, 57%)				45% (31%, 60%)				46% (33%, 60%)	

## Data Availability

The study used publicly available data that can be accessed upon permission from the DHS Program/ICF International.
